# A Phenylalanine to Serine Substitution within an *O-*Protein Mannosyltransferase Led to Strong Resistance to PMT-Inhibitors in *Pichia pastoris*

**DOI:** 10.1371/journal.pone.0062229

**Published:** 2013-05-08

**Authors:** Rebecca Argyros, Stephanie Nelson, Angela Kull, Ming-Tang Chen, Terrance A. Stadheim, Bo Jiang

**Affiliations:** GlycoFi Inc., a wholly-owned subsidiary of Merck & Co. Inc., Lebanon, New Hampshire, United States of America; University of Exeter, United Kingdom

## Abstract

Protein *O-*mannosyltransferases (PMTs) catalyze the initial reaction of protein *O-*mannosylation by transferring the first mannose unit onto serine and threonine residues of a nascent polypeptide being synthesized in the endoplasmic reticulum (ER). The PMTs are well conserved in eukaryotic organisms, and *in vivo* defects of these enzymes result in cell death in yeast and congenital diseases in humans. A group of rhodanine-3-acetic acid derivatives (PMTi) specifically inhibits PMT activity both *in vitro* and *in vivo*. As such, these chemical compounds have been effectively used to minimize the extent of *O-*mannosylation on heterologously produced proteins from different yeast expression hosts. However, very little is known about how these PMT-inhibitors interact with the PMT enzyme, or what structural features of the PMTs are required for inhibitor-protein interactions. To better understand the inhibitor-enzyme interactions, and to gain potential insights for developing more effective PMT-inhibitors, we isolated PMTi-resistant mutants in *Pichia pastoris*. In this study, we report the identification and characterization of a point mutation within the Pp*PMT2* gene. We demonstrate that this F664S point mutation resulted in a near complete loss of PMTi sensitivity, both in terms of growth-inhibition and reduction in *O-*mannosylglycan site occupancy. Our results provide genetic evidence demonstrating that the F664 residue plays a critical role in mediating the inhibitory effects of these PMTi compounds. Our data also indicate that the main target of these PMT-inhibitors in *P. pastoris* is Pmt2p, and that the F664 residue most likely interacts directly with the PMTi-compounds.

## Introduction

The attachment of mannose residues to the hydroxy oxygen groups of serine or threonine residues of the nascent polypeptide chains, commonly referred to as *O-*mannosylation, is an evolutionarily conserved form of post-translational modification for many proteins that enter the secretory pathway [1]–[3]. In humans, defects in *O-*mannosylglycan biosynthesis pathways have been linked to congenital disorders like Walker–Warburg syndrome [4], [5]. In yeast, *O-*mannosylation is essential for cell wall integrity and cell viability [6], [7]. Initiation of the yeast *O-*mannosylation process occurs in the endoplasmic reticulum (ER), where a family of well-conserved protein-*O-*mannosyltransferases (PMTs) transfers the initial mannose residue from dolichol-phosphate-mannose (Dol-P-Man) to the serine or threonine residues of the nascent polypeptide chains [3], [8]. Based on levels of sequence relatedness, PMT members can be grouped into three subfamilies, namely the *PMT1*, *PMT2*, and *PMT4* subfamilies. *Saccharomyces cerevisiae*, the organism in which the *O-*mannosylation process has been studied most comprehensively, contains seven PMT genes (*PMT1-7*). *Candida albicans* and *P. pastoris* both have five PMT genes (*PMT1*, *2*, *4*, *5*, and *6*). In order to carry out their enzymatic functions, members of the PMT1 subfamily (i.e. Pmt1p, Pmt5p) needed to form heterodimers with members of the *PMT2* subfamily (Pmt2p, Pmt3p), whereas Pmt4p, the sole member of the PMT4 subfamily, forms a homodimer [9]. *In vitro*, the Pmt1p-Pmt2p heterodimer is responsible for the majority (>80%) of the PMT enzymatic activities [9]. All PMTs are integral ER membrane proteins with multiple trans-membrane domains, and thus far, no three dimensional structural information is available for any PMT. Using topology-sensitive reporters (i.e., fusions with the catalytic domain of histidinol dehydrogenase and *N-*glycosylation scanning), a topology model for ScPmt1p has been proposed by Strahl-Bolsinger and Scheinost [10]. According to this model, Pmt1p contains seven transmembrane domains with the N-terminus in the cytosol and the C-terminus in the ER lumen. Three hydrophilic loops (#1, #3, and #5) are predicted to face the ER lumen, and three hydrophilic loops (#2, #4, and #6) are proposed to situate in the cytosol. The lumenal loop 1 has been suggested to form part of Pmt1p's catalytic site. The lumenal loop 5 is also important for Pmt1p's activity; however, site-specific mutagenesis studies have shown that the loop 5 is not involved in substrate binding, nor is it required for hetero-dimerization [11], [12]. Because other PMT family members share identical hydropathy profiles to Pmt1p, it is predicted that this topology model may apply to other PMT proteins as well.

In 2004, Orchard *et al.* reported the identification of a group of chemical compounds that inhibit PMT enzymatic activity *in vitro* [13]. These compounds are rhodanine-3-acetic acid derivatives and they were shown to predominantly inhibit Pmt1p in *C. albicans* [13], [14]. However, in *S. cerevisiae*, they seem to block PMT activities of all three subfamily members [15]. Characterizations of the PMT gene family in *P. pastoris* also suggested that these rhodanine-3-acetic acid derivatives affected multiple PMT members, with preference to *PMT1* and *PMT2* gene products [16]. Since their initial publication, these PMT inhibitors have been successfully used by different groups for transcriptional profiling studies in *S. cerevisiae* and *C. albicans* [14], [15], as well as for reducing *O-*mannosylglycan modifications on heterologous proteins expressed in *Ogataea minuta* and *P. pastoris* [17]–[20].

Glycoengineered *P. pastoris* strains, which are capable of producing recombinant proteins with human-like *N-*linked glycosylation, have become very attractive hosts for the production of therapeutic proteins [21], [22]. In addition to *N-*glycosylation, many recombinant proteins expressed in *P. pastoris* and other yeasts are also subject to *O-*mannosylation whereby Ser and/or Thr residues are decorated with mannoses in chain lengths ranging from one to five [2], [3]. These *O-*mannosylglycans may interfere with heterologous protein expression, folding, stability and, more importantly, could give rise to altered pharmacokinetic profiles due to increased clearance [23]. Therefore, to produce safe and efficacious therapeutic proteins in yeast, the host *O-*mannosylation process needs to be tightly controlled. For this purpose, we have identified several chemical analogs with much improved PMT-inhibition potency, and have routinely used them in our fermentation processes to maximally reduce *O-*mannosylglycan modifications on our heterologously expressed protein products [24]. However, despite their diverse applications in both academic and industrial settings, not much is known about the precise mode-of-action of these PMT inhibitors. For example, it is not clear whether these chemical inhibitors interact with the PMT proteins directly and, if so, to which region(s) of the PMT enzymes do these inhibitors bind. To better understand how these PMT inhibitors work, we set out to identify and characterize PMTi-resistant mutants in *P. pastoris*. In this study, we report the identification and characterization of a point mutation within the *PMT2* gene. We demonstrate that this F664S point mutation resulted in a near complete loss of PMTi susceptibility, both in terms of growth-inhibition and *O-*mannosylglycan site occupancy reduction. Our results provided strong genetic evidence indicating that the PMT-inhibitors directly interact with Pmt2p, and that the region surrounding the F664 residue likely plays an important role in PMT function.

## Materials and Methods

### Isolating PMTi-Resistant Mutants by UV Mutagenesis

UV mutagenesis was performed as described previously [25]. Briefly, *P. pastoris* strain y19376 was grown in 40 ml YSD (1% yeast extract, 2% soytone, 2% dextrose) liquid medium overnight at 24°C. Upon reaching an OD600 of 5, a 10 mL aliquot of culture was transferred into an empty 100 mm sterile Petri dish and treated, with the lid off, with 12 mJ/cm^2^ of UV irradiation using a Stratagene UV Stratalinker 2400 (Agilent, California, USA). After the UV treatment, the Petri dish was immediately covered with aluminum foil to prevent photo-induced DNA repair and the mutagenized cells were allowed to recover at 24°C for 3 hours in the dark. Two mL of the recovered y19376 was then centrifuged at 2000 rpm for 5 min in a SORVALL Legend XTR centrifuge (Thermo Scientific USA). The cell pellet was then re-suspended in 400 µL of 2% BMGY (2% Glycerol, 1% yeast extract (YE), 2% peptone, 0.34% yeast nitrogen base w/o amino acids and ammonium sulfate (YNB), 1%(NH_4_)_2_SO_4_ (w/v) and 4×10^5^% biotin in pH 6.0 100 mM potassium phosphate buffer) media, and subsequently plated onto YSD agar plates containing 1 µg/mL, 2 µg/mL, and 4 µg/mL of PMTi inhibitor. After a 7-day incubation at 24°C, colonies were picked and re-streaked onto fresh PMTi-containing plates. Only the clones that displayed a continued PMTi-resistance were kept for further evaluation as PMTi-resistant mutants.

### Growth Inhibitory Curve Determination

Early stationary phase cultures of each strain were first diluted in fresh YSD liquid media to OD600 of 0.05. Subsequently, 400 microliters of the diluted cell suspensions were transferred into a 96-deep-well plate, with each well containing a final concentration series of 100, 33.3, 11.1, 3.7, 1.2, 0.4, 0.14, 0.046, 0.015, 0.005, 0.0017, and 0 µg/ml of either PMTi-3 or PMTi-4 inhibitor. These PMTi-containing cultures were then incubated at 24°C in a shaking incubator (INFORS Multitron, Basel, Switzerland) at 840 rpm, and after 32 hours of growth, the OD600 values were determined for each culture. Percent growth inhibition was defined as [OD600 at the particular PMTi concentration]÷[OD600 at 0 µg/ml PMT-inhibitor]×100.

### Mating and Sporulation of PMTi-Resistant Mutants with a PMTi-Sensitive Strain

To generate diploid strains, zeocin-resistant y17156 and y17157 were mated with y19661 (arsenite-resistant) as previously described [26]. Briefly, strains were grown in 15 mL YSD medium overnight at 24°C. The next day (day 2), the dilution factor was calculated for 50 mL of YSD culture to reach mid-log phase the following day (OD of 0.1–0.8 required for optimal mating efficiency) and cells were diluted. On day 3, approximately 5×10^7^ cells from each strain were mixed in a 50 mL Falcon tube for each mating reaction and then collected on the membrane surface of a vacuum filtration apparatus (MF-MilliporeTM HAWP, mixed cellulose esters, hydrophilic, 0.45 µm pore, 47 mm diameter). Each filter was transferred with cells facing up, to a mating agar plate (0.5% sodium acetate, 1% KCl 1% glucose, 2% agar) and incubated for 5 days at 24°C. The mating reaction was stopped on day 8 by transferring each filter to a 50 mL Falcon tube, washing the mating pairs off with 4 mL YSD. Cells were incubated in a rotating shaker for 3 h at 24°C. The cells were then plated onto selective plates (YSD with 100 µg/ml zeocin and 0.5 mM arsenite) to select for zeocin-resistant and arsenite-resistant diploid strains.

To make clones haploid, sporulation was performed. In preparation for sporulation, positive (diploid) mated clones were patched onto YSD plates and incubated at 24°C for 3 days. Thereafter, cells were patched onto a sporulation plate (0.5% sodium acetate, 1% KCl 1% glucose, 2% agar) and incubated for 4 days at 24°C. Subsequently, cells were scraped off and resuspended in 700 µL diH2O in 1.5 mL Eppendorf tube for each mated clone. For each tube, 700 µL of diethyl ether was added, the mixture was vortexed for 5 min and centrifuged at 6000 rpm for 1 min in a SORVALL Legend XTR centrifuge (Thermo Scientific USA). Approximately 1 mL of liquid was removed from the top, leaving ∼0.5 mL diH2O and the haploid cell pellet. The cell pellet was resuspended in the remaining diH2O. 50 µL and 200 µL of the sporulation mixture was plated out onto YSD plates and incubated at 24°C. To determine if clones retained PMTi resistance, the sporulated clones were replica plated onto YSD plates containing 4 µg/mL PMTi.

### *PMT2* (F664S) Gene Replacement Plasmid Construction

Plasmid pGLY5931 is an integration vector that targets the *PMT2* locus and contains the *P. pastoris URA5* gene flanked by a pair of direct lacZ repeat sequences. On one side of the lacZ-URA5-lacZ cassette is a DNA fragment containing the open reading frame (ORF) encoding the *PMT2* (F664S) point mutation, and on the other side by a DNA fragment from the 3′ region of the *PMT2* gene. After SfiI digestion, the linearized pGLY5931 plasmid is transformed into a ura^−^ *P. pastoris* strain. By double-crossover homologous recombination, the SfiI fragment of the pGLY5931 vector will delete the wild-type *PMT2* ORF and replace it with the mutated *PMT2* (F664S) gene.

### *O-*Mannosylglycan Site Occupancy Determination of Monoclonal Antibody (mAb)

*O-*mannosylglycan site occupancy was quantified using the high-performance anion exchange chromatography with pulsed amperometric detection (HPAED-PAD) method described by Stadheim *et al.* [27]. Monoclonal antibodies (mAbs) were purified by protein A affinity chromatography. The attached *O-*mannose chains were: 1) chemically cleaved from the mAbs using alkaline elimination; 2) hydrolyzed into monosaccharide units (i.e. mannose and mannitol); 3) assayed by HPAEC-PAD to quantify the levels of the monosaccharide residues released from the mAb molecules. The alkaline elimination reaction converts, exclusively, the reducing terminal mannose (i.e. the mannose molecule attached to the Serine or Threonine residue) to a mannitol molecule. Therefore, there is a strict 1:1 relationship between mannitol molecules and the *O-*mannose chain numbers, regardless of the chain-lengths of the mannosylglycans. Determination of the average number of mannitol groups per mAb (H_2_L_2_) molecule gives a precise and accurate assessment of the *O-*mannosylation site occupancy.

### Other Experimental Methods

Yeast transformations were performed using the standard electroporation method as described by Cregg *et al.* [28]. *P. pastoris* strains used in this study are listed in Table 1. Fed-batch fermentations, purification of IgG1, as well as all other analytical assays, were performed essentially as previously described [17], [18], [20].

**Table 1:** Strains used in this study.

Name	Description	Reference
y8316	Glyco-engineered strain capable of generating bi-antennary, galactose-terminated complex *N-*glycan structures. Its complete genotype is: *ura5*Δ*::ScSUC2 och1*Δ*::lacZ bmt2*Δ*::lacZ/KlMNN2-2 mnn4L1*Δ*::lacZ/MmSLC35A3 pno1*Δ *mnn4*Δ*::lacZ ADE1::lacZ/NA10/MmSLC35A3/FB8 his1*Δ*::lacZ/ScGAL10/XB33/DmUGT arg1*Δ*::HIS1/KD53/TC54 bmt4Δ::lacZ bmt1*Δ*::lacZ bmt3*Δ*::lacZ-URA5-lacZ PRO1::ARG1/AOX1p-TrMDS1*	[18]
y19376	Same as y8316, except with the addition of *pmt4*Δ	this study
y17156	UV-mutagenenized, PMTi-resistant mutant #1 derived from y19376	this study
y17157	UV-mutagenenized, PMTi-resistant mutant #2 derived from y19376	this study
y19661	Same as y8316, except with the addition of *URA6::LmSTT3D+ScARR3*	this study
y28432	Same as y8316, except with the addition of *pmt2*Δ*::PMT2 (F664S)*	this study

Abbreviation descriptions.

*ScSUC2*: *S. cerevisiae* Invertase.

*OCH1*: Alpha-1,6-mannosyltransferase.

*KlMNN2-2*: *K. lactis* UDP-GlcNAc transporter.

*BMT1-4*: Beta-mannose-transferase genes 1 to 4.

*MNN4L1*: MNN4-like gene 1.

*MmSLC35A3*: Mouse homologue of UDP-GlcNAc transporter.

*PNO1*: Phosphomannosylation of N-linked oligosaccharides.

*MNN4*: Mannosyltransferase (charge elimination).

*ScGAL10*: UDP-glucose 4-epimerase.

XB33: Truncated *HsGalT1* fused to *ScKRE2* leader.

*DmUGT*: UDP-Galactose transporter.

KD53: Truncated *DmMNSII* fused to *ScMNN2* leader.

TC54: Truncated *RnGNTII* fused to *ScMNN2* leader.

NA10: Truncated *HsGNTI* fused to *PpSEC12* leader.

FB8: Truncated *MmMNS1A* fused to *ScSEC12* leader.

*TrMDS1*: Secreted *T. reseei MNS1*.

*STT3D*: oligosaccharyltransferase.

## Results

### Isolation of PMTi-Resistant Mutants

**Figure 1:**
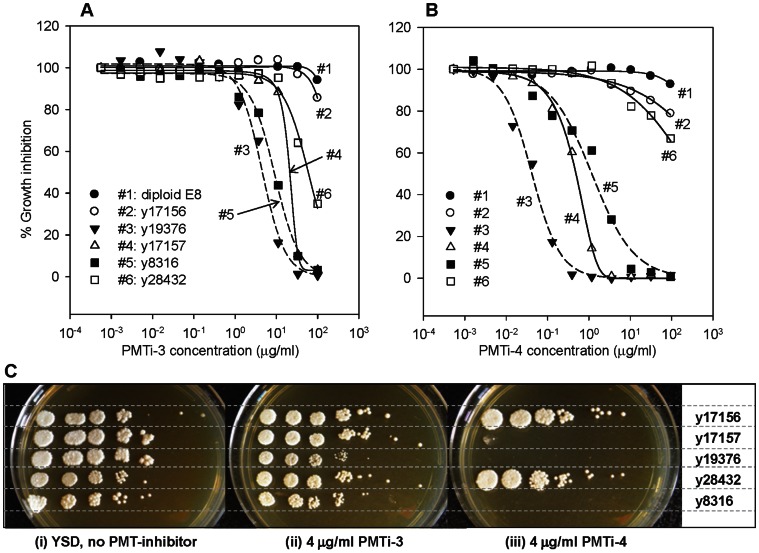
Growth inhibitory effects of PMTi-3 and PMTi-4. Growth inhibitory curves (A, B) and serial dilution spot assay results (C) are shown. The percentages of growth inhibition by the PMTi-3 (A) and PMTi-4 (B) are plotted and curve-fitted using SigmaPlot. The values displayed in the figure were averages from at least 2 independent experiments. The inhibition curves for the pmt4Δ strain (y19376) and the wild type strain (y8316) are shown as dashed-lines, and the others are displayed as solid-lines. For the serial dilution assay (C), overnight-grown saturated cultures of each strain were serial diluted 1∶10, spotted onto YSD, YSD+PMTi-3, and YSD+PMTi-4 plates, and photographed after 72 hours' of growth at 24°C.

PMT inhibitors are rhodanine-3-acetic acid derivatives originally identified as *in vitro* inhibitors against CaPmt1p [13]. To better understand their growth inhibitory effects on *P. pastoris*, we evaluated the susceptibility of two different *P. pastoris* strains against two different PMT inhibitors. The two strains are y8316, which has all the PMT genes (*PMT1,2,4,5,6*) intact, and y19376, which is a pmt4Δ single mutant. The first inhibitor, PMTi-3 (5-[[3-(1-Phenyl-2-hydroxy)ethoxy)-4-(2-phenylethoxy)]phenyl]methylene]-4-oxo-2-thioxo-3-thiazolidineacetic Acid), is one of the original rhodanine-3-acetic acid derivatives described by Orchard *et al.* [13]. The second inhibitor, PMTi-4, is a closely-related chemical analog [24]. As shown in Figure 1, the PMTi-4 inhibitor exhibited much lower IC50 values (y8316: 1 µg/ml; y19376: 0.04 µg/ml) than those of PMTi-3 (y8316: 9 µg/ml; y19376: 5 µg/ml), demonstrating that PMTi-4 is a more potent inhibitor than PMTi-3. Furthermore, these data also illustrated that the pmt4Δ strain (y19376) is hypersensitive to both PMTi-3 and PMTi-4. However, the degree of hypersensitivity varied dramatically. With respect to PMTi-3, the IC50 of y19376 was only 1.8-fold lower than the IC50 of y8316. However, with PMTi-4 the IC50 value of y19376 dropped 25-fold, suggesting that the pmt4Δ strain was significantly more sensitive to PMTi-4. In order to minimize background cell growth and to maximize the sensitivity and selectivity for our genetic selection, we decided to perform our mutagenesis and PMTi-resistance selection using the hypersensitive y19376 (pmt4Δ) host and the potent PMTi-4 inhibitor.

To better understand the molecular mechanism of PMT-inhibition, and to gain potential insights for developing more effective PMT-inhibitors, we decided to identify PMTi-resistant mutants in *P. pastoris*. To this end, we randomly mutagenized y19376 by UV irradiation, and subjected the mutagenized cells to growth-inhibitory concentrations of PMTi-4. From approximately 10^7^ UV-treated cells, we identified two independent mutants: y17156 and y17157. The y17156 mutant appeared to be extremely resistant to PMTi-4, increasing the IC50 by more than 5000-fold (from 0.02 µg/ml to greater than 100 µg/ml). In comparison, y17157 displayed a modest, but easily detectable improvement in its tolerability to PMTi-4 with a 25-fold increase in its IC50 values.

**Figure 2:**
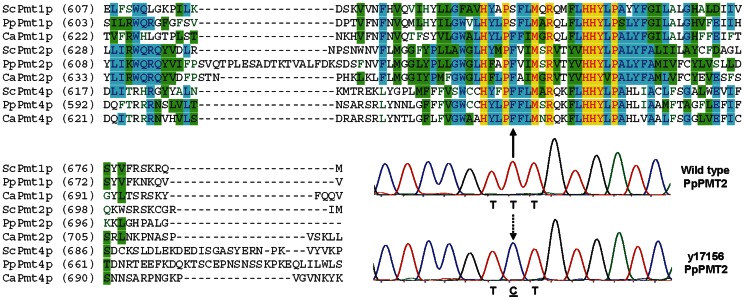
Identification of a point mutation within a highly conserved region of PpPmt2p. Sequence alignments of the predicted cytosolic loop #6 [11], and the sequencing traces displaying the T to C mutation identified in the PMTi-resistant mutant (y17156). Amino acid residues identical in all PMT sequences are highlighted in yellow, residues identical in more than half of the sequences are highlighted in blue, and conserved residues are highlighted in green. The dashed down arrow indicates the T to C nucleotide exchange caused by the UV-mutagenesis, and the solid up arrow pointed to the position of the resulting F to S amino acid substitution in the protein sequence of the PpPmt2p enzyme.

A common mechanism for microbial organisms to become resistant to anti-microbial agents is to acquire mutations in the target proteins against which the anti-microbial compounds were directed, so as to prevent or minimize the compound-target interactions [29]. For example, it has been well documented that many fluconazole-resistant, or caspofungin-resistant *C. albicans* strains isolated from patients contained mutations in *ERG11*, or *FKS1*, the molecular targets of fluconazole or caspofungin, respectively [30], [31]. To examine if any of the putative PMTi target genes were mutated, we sequenced *PMT1*, *2*, *5*, and *6* in both PMTi-resistant mutants. No mutations were found in any of these PMT genes for y17157, demonstrating that the resistance mechanism of this mutant did not involve mutations in the *PMT* genes. Since overexpression of efflux pumps to minimize intracellular drug accumulation is another common mechanism for drug resistance [29], it will be of interest to examine if drug efflux pumps were up-regulated in the y17157 mutant. In contrast to y17157, we did identify a single T to C nucleotide mutation in the *PMT2* coding region for y17156, the strain that exhibited the very strong PMTi-4 resistant phenotype (Figure 2). For the rest of this manuscript, we will only focus on y17156, and leave the characterization of the other mutant y17157 for another communication.

The T to C mutation resulted in an amino acid substitution from Phe to Ser at residue 664, which is located within a large loop predicted to face the cytosol [10]. As shown in Figure 2, amino acids surrounding this phenylalanine are highly conserved among members of all the PMT sub-families, suggesting that this area is most likely to be functionally important.

To determine whether PMTi-resistance was dominant or recessive, we crossed y17156 with a sensitive, and non-mutagenized, strain y19661, and examined the diploid for its susceptibility to the PMTi-inhibitors. Interestingly, the diploid strain displayed an IC50 value comparable to the haploid y17156 for both PMTi-3 and PMTi-4, demonstrating that the diploid strain retained the strong PMTi-resistant phenotype. This suggests that the PMTi-resistant phenotype of y17156 is fully dominant. To evaluate the connection between this PMTi-resistant phenotype and the observed F664S mutation, we performed a random-spore analysis on the diploid strain, and followed the segregation patterns of pmt4Δ, LmSTT3D (*Leishmania major* STT3D gene encoding the D subunit of oligosaccharyltransferase complex) [32], PMTi-resistance, and the F664S mutation. As shown in Table 2, the PMTi-resistance phenotype segregated independently from both pmt4Δ and STT3D. However, with the 6 random spores we examined, the PMTi-resistance phenotype always co-segregated with the F664S mutation. This co-segregation pattern strongly suggests that the *PMT2* F664S mutation might be the causative mutation responsible for the PMTi-resistant phenotype.

**Table 2:** Segregation patterns of the PMTi-resistant phenotype, the F664S mutation, and other genetic markers.

		PMT4 ORF	STT3D ORF	Resistance to PMT inhibitor	PMT2 Sequencing
1	yGLY17156 haploid	−	−	+	F664S
2	yGLY19661 haploid	+	+	−	wild type
3	Diploid (yGLY17156×yGLY19661)	+	+	+	not determined
4	Random Spore A	−	+	+	F664S
5	Random Spore B	+	+	+	F664S
6	Random Spore C	−	+	+	F664S
7	Random Spore D	−	+	+	F664S
8	Random Spore E	−	+	−	wild type
9	Random Spore F	−	−	−	wild type

### The *PMT2*-F664S Point-Mutation is Sufficient for PMTi-Resistance

**Figure 3:**
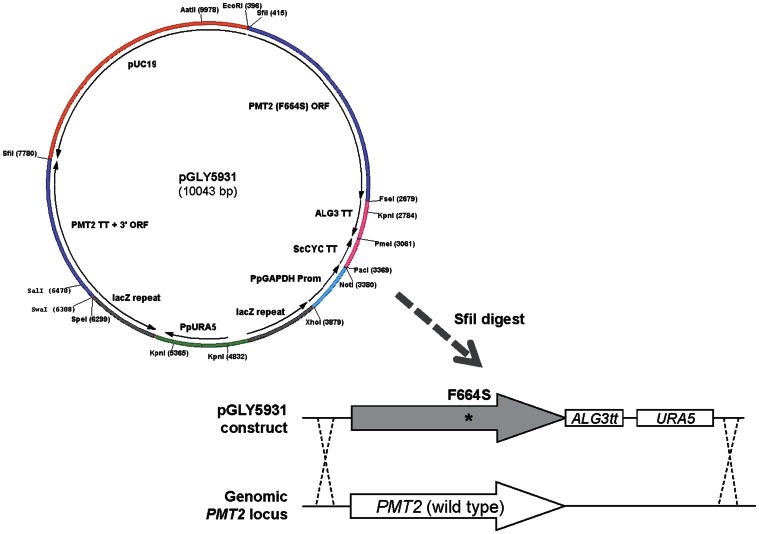
Construction of PMT2 (F664S) mutant by gene targeting and replacement. Plasmid map of pGLY5931 and schematic representation of the DNA construct used to replace the endogenous *PMT2* ORF with the F664S mutant version. The “*” indicated the approximate location of the F664S mutation within the ORF.

To evaluate if the identified *PMT2*-F664S point-mutation is responsible for the observed PMTi-resistance phenotype, we constructed a DNA construct, pGLY5931, which enabled us to specifically delete the wild-type *PMT2* gene, and replace it with the F664S mutant version in a given *P. pastoris* strain (Figure 3). As expected, we observed that the targeted replacement of the F664S mutation successfully changed the PMTi-susceptible hosts into strains that are highly resistant to PMTi inhibitors. As shown in Figure 1, the *PMT2*-F664S point mutation increased the PMTi-4 IC50 values from ∼1 µg/ml (y8316) to over 100 µg/ml (y28432). For PMTi-3, the IC50 values increased from 9 µg/ml to 59 µg/ml. These results demonstrated that the *PMT2*-F664S point mutation alone is sufficient for causing the PMTi-resistant phenotype. Furthermore, identification of the PMTi-resistant F664S substitution within the PMT2 gene strongly suggested a direct interaction between the PMTi inhibitor and the Pmt2p protein, most likely within a region very close to the F664 residue.

### Effects of the *PMT2*-F664S Mutation on *O-*Mannosylation

To further evaluate how the *PMT2*-F664S mutation affects the *O-*mannosylation process, we examined *O-*mannosylglycan modifications of two different recombinant monoclonal antibodies (mAbs) known to be *O-*mannosylated in yeast. These mAbs were expressed in a *P. pastoris* host that contained the F664S *PMT2* gene. To this end, we utilized 1L-scale bioreactors to express both mAbs, using a standard *P. pastoris* fermentation process in the presence, or absence, of the PMTi-4 inhibitor. Upon completion of the fermentation, secreted mAbs were purified from the supernatant by protein A affinity chromatography, and then subjected to *O-*mannosylglycan site occupancy (average number of mannose chains attached to each mAb H_2_L_2_ molecule) and product titer determinations.

**Figure 4:**
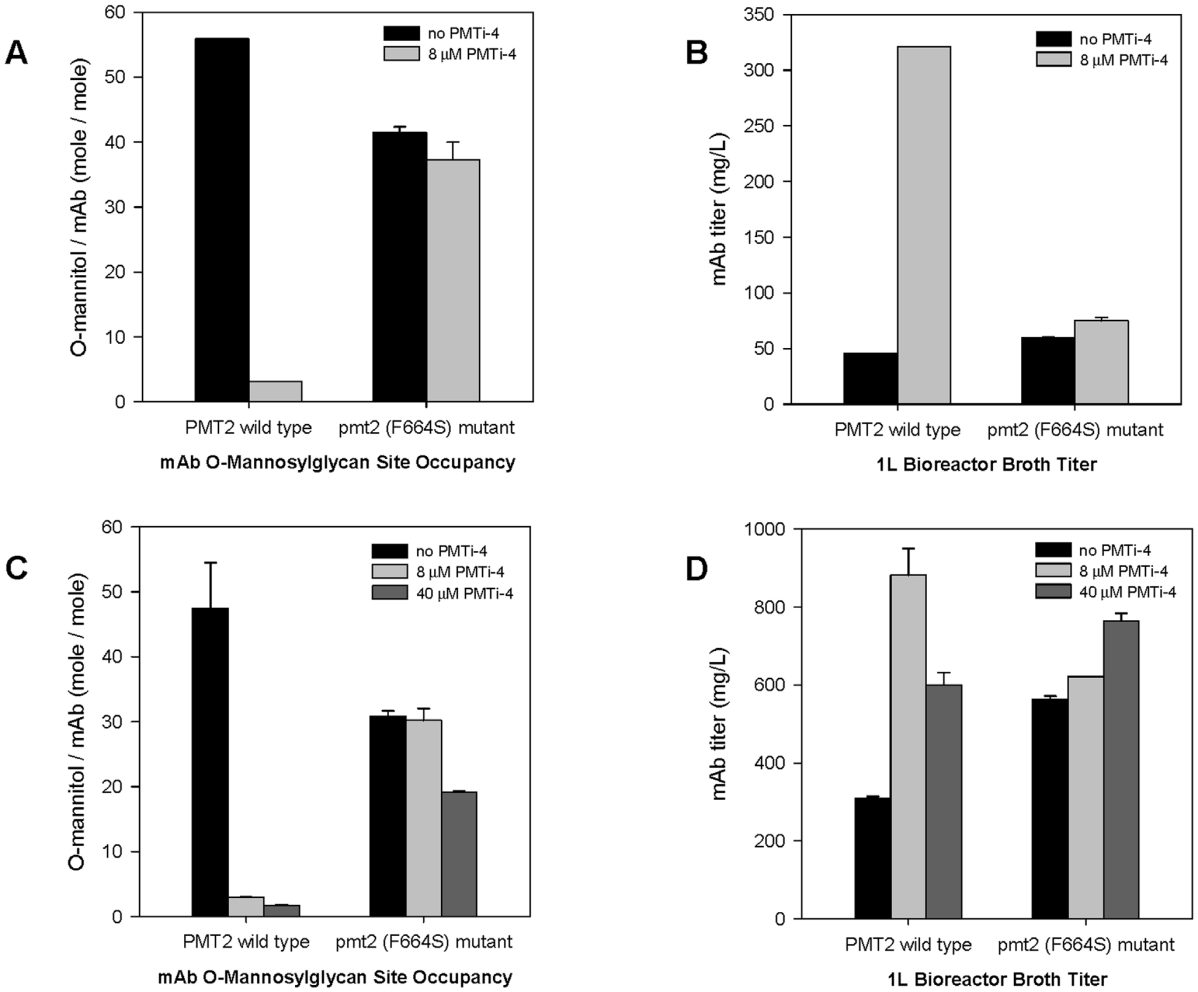
Effects of the *PMT2* (F664S) mutation on *O-*mannosylglycan site occupancy and product titer. *O-*mannosylglycan site occupancy (A and C) and product titers (B and D) were determined for mAbs purified from *PMT2* wild type or pmt2 (F664S) mutant strains. Panels A and B displayed results for mAb#1 expressed with 0 or 8 µM PMTi-4 inhibitor; and panels C and D showed resulted for mAb#2 produced in the presence of 0, 8, or 40 µM of PMTi-4. Results are shown as average ± stdev derived from at least two independent experiments.

In absence of PMTi-4, mAbs produced from strains with wild type *PMT2* were heavily *O-*mannosylated with an average of 56 and 48 *O-*mannosylation events per each H_2_L_2_ molecules of mAb#1 and #2, respectively (Figure 4A and 4C). From the F664S mutant strain background, the *O-*mannosylglycan site occupancy of mAb#1 and #2 were 42 and 31, reflecting approximately 35% and 26% reductions from the *O-*mannosylglycan site occupancies observed for the corresponding *PMT2* wild type hosts. Because a pmt2Δ null mutant displayed a much more pronounced reduction (∼87%) in *O-*mannosylglycan site occupancy [16], results shown in Figure 4A and 4C suggested that the F664S point mutation resulted in a partial decrease in the *in vivo* activity of the mutated Pmt2p (F664S) protein. In the presence of PMTi-4, the inhibitor dramatically reduced the *O-*mannosylglycan site occupancy for mAbs derived from the *PMT2* wild type strains (from 56 to 3.1 for mAb#1, and 48 to 2.9 for mAb #2). Increasing the PMTi-4 dose (5-fold higher) reduced the *O-*mannosylglycan site occupancy slightly further to 1.8 for mAb#2. In contrast, PMTi-4 inhibitor exhibited very limited effects in strains containing the *PMT2*-F664S mutation with *O-*mannosylglycan site occupancy moving from 42 and 31 to 37 and 30 for mAb#1 and #2, respectively. With 5-times of the normal PMTi-4 dosage, mAb#2 produced in the F644S mutant strain remained extensively *O-*mannosylated, with an *O-*mannosylglycan site occupancy of 19 per molecule of mAb. These results demonstrated that the F664S mutation led to a near complete loss of susceptibility to the inhibitory effects of PMTi-4. Interestingly, we also observed a clear negative correlation between *O-*mannosylglycan site occupancy and mAb product titer (Figure 4B and 4D). The addition of PMTi-4 increased the production titer of the *PMT2* wild type strains, from 46 to 321 mg/L for mAb#1, and from 321 to 881 mg/L (1x PMTi-4 dosage), and to 600 mg/L (5x PMTi-4 dosage) for mAb#2. For strains containing the F664S mutation, PMTi-4 addition had a much less profound impact on product titer changing from 60 to 75 mg/L for mAb#1, and from 563 to 622 mg/L with 1x PMTi-4 dosing, and to 765 mg/L with 5x PMTi-4 dosing for mAb#2. It is of interest to note that in the absence of PMTi-4 inhibition, strains with the F664S mutation displayed higher product titers than the corresponding *PMT2* wild type hosts, with 30% and 75% increase for mAb#1 and #2, respectively.

## Discussion

PMTs play essential roles in the *O-*mannosylation process. There are up to seven PMT genes encoding Pmt homologues in yeast, and the Pmt1p-Pmt2p heterodimer accounts for greater than 80% of the PMT enzymatic activities *in vitro* [9]. In this study, we report the identification of the *PMT2*-F664S substitution as the causative mutation for the PMTi-resistant phenotype, and report the characterization of the effects of this mutation on the *O-*mannosylation of selected monoclonal antibodies expressed in these PMTi-resistant host strains.

Mutant y17156 displayed an extremely strong PMTi-resistant phenotype: the UV-mutagenesis has led to a greater than 5000-fold increase in the IC50 values against the PMTi-4 inhibitor. As a matter of fact, we were unable to determine the minimum inhibitory concentration (MIC) for y17156, because at the highest PMTi-4 concentration tested (100 µg/ml), it still exhibited very robust growth. Because acquiring mutations within the target gene is one of the most common mechanisms for pathogenic fungal organisms to become resistant to anti-fungal drugs [29], [33], it was not totally unexpected for us to identify a single amino acid substitution within a mannosyltransferase of the PMT family. The findings that the F664S co-segregated with the PMTi-resistant phenotype, and that introducing the F664S mutation into a PMTi-sensitive strain resulted in strong PMTi-resistance provided strong evidence that the F664S mutation in the *PMT2* gene is the causative mutation for the PMTi-resistance phenotype.

We have shown that, in the absence of PMT-inhibitors, the F664S point mutation led to modest reductions (between 26 to 35%) in the average number of *O-*mannose chains attached on mAbs molecules. However, in the presence of 8 mM PMTi-4, the dosage that effectively eliminated ∼95% of the *O-*mannosylglycan site occupancy in strains containing wild type *PMT* genes, the F664S substitution completely blocked any inhibitory effect the PMTi-4 had on *O-*mannosylation. The *O-*mannosylglycan site occupancies observed from mAbs expressed from the F664S-containing hosts exhibited no detectable changes either without or with 8 mM PMTi-4 inhibitor (41.5+/−0.8 vs. 37.3+/−2.8 for mAb#1; 30.8+/−0.8 vs. 30.2+/−1.8 for mAb#2). Consistent with this lack of reduction in *O-*mannosylglycan site occupancy levels, strains harboring the F664S mutation became extremely resistant to the growth inhibitory effects of PMTi-4 with IC50 values increasing to at least 100-fold in the wild type strain background, and over 5000-fold in the pmt4Δ strain background. Collectively, these results suggested that the F664S point mutation led to a partial loss of the enzymatic function of Pmt2p, and simultaneously resulted in a near complete loss of susceptibility to PMTi-4 inhibition. Furthermore, our data provided strong genetic evidence that, in *P. pastoris*, the PMTi-4 compound inhibited the *O-*mannosylation process predominantly through Pmt2p inactivation, and the observed strong PMTi-resistant phenotype is a direct consequence of the F664S mutation preventing or disrupting the Pmt2p-PMTi-4 interactions. Lastly, our results also indicated that the Pmt2p enzyme, or more precisely the Pmt1p-Pmt2p heterodimer complex, is responsible for the majority of *O-*mannosylglycan modifications for mAbs heterologously expressed from *P. pastoris* strains, because inhibition of the Pmt2p enzyme by PMTi-4 blocked most of the *O-*maanosylglycan modifications on the mAb molecules.

*PMT* genes are very well conserved from yeast to human. Members of the *PMT1*, *2*, and *4* subfamilies are all multi-span integral membrane proteins, sharing very similar hydropathy profiles. Multi-span membrane proteins are very difficult to isolate in sufficient quantities and purity to permit crystallization. Consequently, like most multi-span membrane proteins, no three dimensional structures of any PMT enzymes have been obtained so far. Therefore, very little is understood about the active-site composition and substrate-binding characteristics of PMT-family enzymes, the structural elements participating in PMTi interactions, and how these inhibitor-protein interactions translate into enzymatic inactivation. To our knowledge, results reported in this study provide the first experimental evidence indicating that the region surrounding amino acid F664 participates in the interaction between the PMT-inhibitors and the Pmt2p enzyme. Based on the topology model proposed by Strahl-Bolsinger and Scheinost [10], the F664 residue is located within a large hydrophilic loop (loop#6) facing the cytosol. The amino acid residues surrounding F664 are highly conserved among all PMT subfamily members. Previous site-directed mutagenesis studies on ScPmt1p has implicated that the C-terminal part of the loop#6 might be required for either Pmt1p-Pmt2p hetero-dimerization and/or Pmt1p enzymatic activity, because a truncated ScPmt1p containing approximately half of loop#6 failed to dimerize with Pmt2p, and also lost its enzymatic function [11]. Identification of the F664 residue as a critical amino acid required for PMTi-4 inhibition of the Pmt2p enzyme provides further evidence supporting the notion that this cytosolic loop plays an important role in Pmt2p's *in vivo* function.

PMTi-4 is derived, by medicinal chemistry modifications, from the structurally-related PMTi-3 (5-[[3-(1-Phenyl-2-hydroxy)ethoxy)-4-(2-phenylethoxy)]phenyl]methylene]-4-oxo-2-thioxo-3-thiazolidineacetic Acid). It has been shown that, in *C. albicans*, PMTi-3 inactivated the CaPmt1p enzyme *in vitro* [13], [14]. Gene deletion analyses of the *PMT* gene family in *P. pastoris* suggested that PMTi-3 preferentially inhibited *PpPMT1* and *PpPMT2* gene products *in vivo* [16]. In *S. cerevisiae* PMTi-3 did not appear to have any preference to any *PMT* genes, rather, it broadly inhibited all PMT family members quite equally. Although the *PMT2*-F664S mutation resulted in significant resistance to both PMTi-3 and PMTi-4 inhibitors, it is worth noting that the F664S mutation responded to inhibitions by PMTi-3 or by PMTi-4 quite differently. For strains containing intact PMT genes (i.e., y8316), the PMTi-4 compound is a much more potent inhibitor than PMTi-3 (Figure 1). However, for the F664S-containing strain (y28432), the PMTi-3 inhibitor became slightly more potent than PMTi-4: with IC50 values of ∼60 versus >100 µg/ml. This interesting reversal of potency between the two closely-related inhibitors indicated that the F664S substitution disrupted the Pmt2p-PMTi-4 interactions more effectively than the Pmt2p-PMTi-3 interaction; and suggested that the F664 residue might preferentially and directly interact with structural elements only present in the PMTi-4 molecule. We cannot rule out the possibility that the F664 residue interacted with the PMTi-4 compound indirectly, either via a different area of the Pmt2p protein or through an unidentified “third-party” component. More detailed genetic and biochemical characterizations are needed to formally distinguish whether or not PMTi-4 binds Pmt2p directly, to evaluate whether other residues surrounding F664 are also involved in PMTi-4 interaction and to elucidate the mechanism for how the interaction between PMTi-4 and a cytosolic loop led to a dramatic inactivation of Pmt2p, whose active-site is located in the ER lumen.

## References

[1] Spiro RG (2002) Protein glycosylation: nature, distribution, enzymatic formation, and disease implications of glycopeptide bonds. Glycobiology 12: 43R–56R. 10.1093/glycob/12.4.43r 12042244

[2] Goto M (2007) Protein O-glycosylation in fungi: diverse structures and multiple functions. Biosci Biotechnol Biochem 71: 1415–1427. 17587671 10.1271/bbb.70080

[3] Lommel M, Strahl S (2009) Protein O-mannosylation: conserved from bacteria to humans. Glycobiology 19: 816–828. 19429925 10.1093/glycob/cwp066

[4] Beltrán-Valero de Bernabé D, Currier S, Steinbrecher A, Celli J, van Beusekom E, et al (2002) Mutations in the O-mannosyltransferase gene POMT1 give rise to the severe neuronal migration disorder Walker-Warburg syndrome. Am J Hum Genet 71: 1033–1043. 12369018 10.1086/342975 PMC419999

[5] Lehle L, Strahl S, Tanner W (2006) Protein glycosylation, conserved from yeast to man: a model organism helps elucidate congenital human diseases. Angew Chem Int Ed Engl 45: 6802–6818. 17024709 10.1002/anie.200601645

[6] Gentzsch M, Tanner W (1996) The PMT gene family: protein O-glycosylation in Saccharomyces cerevisiae is vital. Embo J 15: 5752–5759. 8918452 PMC452322

[7] Willer T, Brandl M, Sipiczki M, Strahl S (2005) Protein O-mannosylation is crucial for cell wall integrity, septation and viability in fission yeast. Mol Microbiol 57: 156–170. 15948957 10.1111/j.1365-2958.2005.04692.x

[8] Ernst JF, Prill SK (2001) O-glycosylation. Med Mycol 39 Suppl 1: 67–74. 11800270

[9] Girrbach V, Strahl S (2003) Members of the evolutionarily conserved PMT family of protein O-mannosyltransferases form distinct protein complexes among themselves. J Biol Chem 278: 12554–12562. 12551906 10.1074/jbc.M212582200

[10] Strahl-Bolsinger S, Scheinost A (1999) Transmembrane topology of pmt1p, a member of an evolutionarily conserved family of protein O-mannosyltransferases. J Biol Chem 274: 9068–9075. 10085156 10.1074/jbc.274.13.9068

[11] Girrbach V, Zeller T, Priesmeier M, Strahl-Bolsinger S (2000) Structure-function analysis of the dolichyl phosphate-mannose: protein O-mannosyltransferase ScPmt1p. J Biol Chem 275: 19288–19296. 10764776 10.1074/jbc.M001771200

[12] Lommel M, Schott A, Jank T, Hofmann V, Strahl S (2011) A conserved acidic motif is crucial for enzymatic activity of protein O-mannosyltransferases. J Biol Chem 286: 39768–39775. 21956107 10.1074/jbc.M111.281196 PMC3220539

[13] Orchard MG, Neuss JC, Galley CM, Carr A, Porter DW, et al (2004) Rhodanine-3-acetic acid derivatives as inhibitors of fungal protein mannosyl transferase 1 (PMT1). Bioorg Med Chem Lett 14: 3975–3978. 15225710 10.1016/j.bmcl.2004.05.050

[14] Cantero PD, Lengsfeld C, Prill SK, Subanović M, Román E, et al (2007) Transcriptional and physiological adaptation to defective protein-O-mannosylation in Candida albicans. Mol Microbiol 64: 1115–1128. 17501932 10.1111/j.1365-2958.2007.05723.x

[15] Arroyo J, Hutzler J, Bermejo C, Ragni E, García-Cantalejo J, et al (2011) Functional and genomic analyses of blocked protein O-mannosylation in baker's yeast. Mol Microbiol 79: 1529–1546. 21231968 10.1111/j.1365-2958.2011.07537.x

[16] Nett JH, Cook WJ, Bobrowicz P, Kett W, Brenova E (2013) Characterization of the Pichia pastoris protein-O-mannosyltransferase gene family. PLOS ONE (submitted). 10.1371/journal.pone.0068325 PMC3698189 23840891

[17] Barnard GC, Kull AR, Sharkey NS, Shaikh SS, Rittenhour AM, et al (2010) High-throughput screening and selection of yeast cell lines expressing monoclonal antibodies. J Ind Microbiol Biotechnol 37: 961–971. 20711797 10.1007/s10295-010-0746-1

[18] Hopkins D, Gomathinayagam S, Rittenhour AM, Du M, Hoyt E, et al (2011) Elimination of beta-mannose glycan structures in *Pichia pastoris* . Glycobiology 21: 1616–1626. 21840970 10.1093/glycob/cwr108

[19] Kuroda K, Kobayashi K, Kitagawa Y, Nakagawa T, Tsumura H, et al (2008) Efficient antibody production upon suppression of O mannosylation in the yeast Ogataea minuta. Appl Environ Microbiol 74: 446–453. 18039826 10.1128/AEM.02106-07 PMC2223252

[20] Potgieter TI, Cukan M, Drummond JE, Houston-Cummings NR, Jiang Y, et al (2009) Production of monoclonal antibodies by glycoengineered *Pichia pastoris* . J Biotechnol 139: 318–325. 19162096 10.1016/j.jbiotec.2008.12.015

[21] Hamilton SR, Davidson RC, Sethuraman N, Nett JH, Jiang Y, et al (2006) Humanization of yeast to produce complex terminally sialylated glycoproteins. Science 313: 1441–1443. 16960007 10.1126/science.1130256

[22] Li H, Sethuraman N, Stadheim TA, Zha D, Prinz B, et al (2006) Optimization of humanized IgGs in glycoengineered *Pichia pastoris* . Nat Biotechnol 24: 210–215. 16429149 10.1038/nbt1178

[23] Li H, d'Anjou M (2009) Pharmacological significance of glycosylation in therapeuticproteins. Curr Opin Biotechnol 20: 678–684. 19892545 10.1016/j.copbio.2009.10.009

[24] Desai R, Yang LH (2009) Efficient production of heterologous proteins using mannosyl transferase inhibitors. WO 2009/143041 A1 (patent application)

[25] Winston F (2008) EMS and UV mutagenesis in yeast. Curr Protoc Mol Biol Chapter 13: Unit 13 3B. 10.1002/0471142727.mb1303bs82 18425760

[26] Chen MT, Lin S, Shandil I, Andrews D, Stadheim TA, et al (2012) Generation of diploid Pichia pastoris strains by mating and their application for recombinant protein production. Microb Cell Fact 11: 91. 22748191 10.1186/1475-2859-11-91 PMC3503796

[27] Stadheim TA, Li H, Kett W, Burnina IN, Gerngross TU (2008) Use of high-performance anion exchange chromatography with pulsed amperometric detection for O-glycan determination in yeast. Nat Protoc 3: 1026–1031. 18546597 10.1038/nprot.2008.76

[28] Cregg JM, Tolstorukov I, Kusari A, Sunga J, Madden K, et al (2009) Expression in the yeast Pichia pastoris. Methods Enzymol 463: 169–189. 19892173 10.1016/S0076-6879(09)63013-5

[29] Peman J, Canton E, Espinel-Ingroff A (2009) Antifungal drug resistance mechanisms. Expert Rev Anti Infect Ther 7: 453–460. 19400764 10.1586/eri.09.18

[30] Morio F, Loge C, Besse B, Hennequin C, Le Pape P (2010) Screening for amino acid substitutions in the Candida albicans Erg11 protein of azole-susceptible and azole-resistant clinical isolates: new substitutions and a review of the literature. Diagn Microbiol Infect Dis 66: 373–384. 20226328 10.1016/j.diagmicrobio.2009.11.006

[31] Balashov SV, Park S, Perlin DS (2006) Assessing resistance to the echinocandin antifungal drug caspofungin in Candida albicans by profiling mutations in FKS1. Antimicrob Agents Chemother 50: 2058–2063. 16723566 10.1128/AAC.01653-05 PMC1479158

[32] Choi BK, Warburton S, Lin H, Patel R, Boldogh I, et al (2012) Improvement of N-glycan site occupancy of therapeutic glycoproteins produced in Pichia pastoris. Appl Microbiol Biotechnol 95: 671–682. 22569635 10.1007/s00253-012-4067-3

[33] Perlin DS (2007) Resistance to echinocandin-class antifungal drugs. Drug Resist Updat 10: 121–130. 17569573 10.1016/j.drup.2007.04.002 PMC2696280

